# 

**Published:** 1869-01

**Authors:** 


### CONTENTS.

ORIGINAL COMMUNICATIONS.

Mechanical Dentistry—Past and Present .......... 9
A Plea for Specialties .......... 14
An Experience .......... 18

PROCEEDINGS OF SOCIETIES.

Report of Discussions at the Ohio State Dental Society, Dec., 1 and 2, 1868 .......... 22

Correspondence .......... 37

SELECTIONS.

Influence of Diet upon the Mother's Milk .......... 40
Dentists in Belgium .......... 40
Preservation of Animal Tissues .......... 41
The Microscope .......... 41
Continuous Electrical Currents in the Treatment of the Suspension of Vital Actions Caused by Chloroform .......... 43
Odontomes .......... 44
Ricord .......... 45
Subperiosteal Resection .......... 46
Test for Pure Glycerine .......... 47
Testing Water for Organic Impurities .......... 47
Was the Nerve Regenerated .......... 48
The Fee for Testifying as Expert .......... 48

EDITORIAL.

Rose Pearl .......... 49
Dental Depot—A Change .......... 50
The Mississippi Valley Dental Association .......... 51
Commencement .......... 51
Floral Guide .......... 52
The Ohio Dental College Association .......... 52

